# Extra Loading Dose of Dexmedetomidine Enhances Intestinal Function Recovery After Colorectal Resection: A Retrospective Cohort Study

**DOI:** 10.3389/fphar.2022.806950

**Published:** 2022-04-25

**Authors:** Guo-Zun He, Ning Bu, Ya-Juan Li, Yuan Gao, Ge Wang, Zhi-Dong Kong, Min Zhao, Shan-Shan Zhang, Wei Gao

**Affiliations:** ^1^ Center for Brain Science, Center for Translational Medicine, Department of Anesthesiology, The First Affiliated Hospital of Xi’an Jiaotong University, Xi’an, China; ^2^ Department of Anesthesiology, Xi’an Aerospace General Hospital, Xi’an, China

**Keywords:** postsurgical recovery, dexmedetomidine, colorectal tumor, systemic inflammation, postoperative gastrointestinal dysfunction

## Abstract

**Importance:** Postoperative gastrointestinal dysfunction (POGD) may be caused by postoperative vagus nerve tension inhibition and systemic inflammation. Dexmedetomidine (Dex) increases vagus nerve tone and affords an anti-inflammatory property, which may play a role in pathogenesis.

**Objective:** To investigate whether a higher dose of Dex enhances gastrointestinal function recovery.

**Design:** In this retrospective study, patients receiving colorectal surgery at the First Affiliated Hospital of Xi’an Jiaotong University from 2017 to 2019 were included. We evaluated the postoperative flatus time between recipients who received loading plus maintenance dose of DEX (LMD group, 237 recipients) and those who recieved maintenance dose of DEX (MD group, 302 recipients). Data were analyzed by logical regression and stratified and interaction analyses. The simulated pharmacokinetics of two DEX regimens was compared using the Tivatrainer software. Thirty paired blood samples from patients whose propensity scores matched with POGD-related factors at 24 h postoperatively were randomly selected, and their tumor necrosis factor-*α* (TNF-α), cyclooxygenase-2 (COX-2), d-lactate (DLA), acetylcholine (Ach), interleukin (IL)-10, lipopolysaccharide (LPS), IL-6, and inducible nitric oxide synthase (iNOS) levels were measured.

**Setting:** Operating rooms and general surgery wards.

**Participants:** Among the 644 patients undergoing colorectal surgery, 12 who had a colostomy, 26 without Dex infusion, 20 whose Dex administration mode cannot be classified, and 47 with a history of intestinal surgery were excluded. A total of 539 patients were included.

**Result:** Compared with the MD group, the LMD group had a shorter recovery time to flatus; lower incidences of nausea, vomiting, abdominal distension, and abdominal pain (*p* < 0.05); and a slightly decreased heart rate. The LMD group was the independent factor of POGD (OR = 0.59, 95% CI = 0.41–0.87, *p* = 0.007) without being reversed in stratified and interaction analyses and had higher Dex plasma concentration from skin incision to 8 h postoperatively. The LMD group had a 39% and 43% increase in Ach and IL-10 levels, respectively, and a 33%–77% decrease in TNF-α, IL-6, COX-2, iNOS, LPS, and DLA levels (*p* < 0.05).

**Conclusion:** Adding an extra loading dose of Dex can increase parasympathetic tone and decrease inflammation; hence, it can enhance postoperative gastrointestinal function recovery following colorectal surgery.

## Introduction

Colorectal cancer ranked as the fourth most prevalent malignant tumor and the fifth leading cause of mortality in 2017 (Yin et al., 2019). Colorectal surgery is the frontline therapy for colorectal cancer, and the surgical outcome is considerably good with a relatively low 5-year tumor recurrence rate of 6.9% (Frontali et al., 2020). Postoperative gastrointestinal dysfunction (POGD), which affects early surgical recovery, commonly occurs after abdominal surgery (Traut et al., 2008; Millan et al., 2012). POGD is a transient impairment of gastrointestinal function after abdominal surgery and is manifested as delayed flatus and defecation, nausea, vomiting, abdominal tenderness, and abdominal distention (Mazzotta et al., 2020). The occurrence of POGD prolongs hospitalization time and increases treatment costs enormously (Vather et al., 2013; Murphy et al., 2016; Gao et al., 2021). Various strategies have been used to prevent POGD, including early postoperative feeding, avoidance of nasogastric tube use, limitation of fluid infusion, the use of minimally invasive surgery, and the use of symptomatic treatment drugs and chewing gum, but their effectiveness is limited because of the lack of pathogenesis-related targeted therapies (Bragg et al., 2015; Wolthuis et al., 2016; Wang et al., 2020; Wattchow et al., 2021).

Surgical stress and systemic inflammation are suggested to be the main causes of POGD (Bragg et al., 2015; Venara et al., 2016). POGD has two phases. In the first phase, neurogenic reflexes suppress splanchnic nerve pathways from skin incision to 3–6 h postoperatively (Stakenborg et al., 2017). In the second phase, innate pro-inflammatory cytokines, such as interleukin-6 (IL-6) and tumor necrosis factor-α (TNF-α), are released because of inflammatory outbursts from 3 h after skin incision to 72 h postoperatively. In addition, other inflammatory mediators, including cyclooxygenase-2 (COX-2) and inducible nitric oxide synthase (iNOS), are increased, whereas anti-inflammatory cytokines, such as interleukin-10 (IL-10), are decreased. All of these changes can impair the intestinal barrier; moreover, lipopolysaccharide (LPS) and d-lactate (DLA) in the gut can be diffused into circulation and further worsen PODG (Farro et al., 2016; Stakenborg et al., 2017).

Dexmedetomidine (Dex) is a highly selective *α*_2_ receptor agonist with sedative, analgesic, anti-anxiety, sympathetic inhibitory, and less respiratory inhibitory effects (Song et al., 2016). As an auxiliary drug for general anesthesia, Dex can activate the vagal efferent system and inhibit inflammation (Wang et al., 2015; Obara 2018; Bao and Tang, 2020). Animal studies showed that the anti-inflammatory effect of Dex is better at a high dose than at a low dose, and the effect of “pre-empty” treatment is better than that of the “post-empty” treatment (Yeh et al., 2016; Bao and Tang, 2020). A clinical research reported that the preoperative infusion of 1 μg/kg Dex for 10 min and the intraoperative continuous infusion of 0.3 μg/kg/h Dex were more beneficial to the recovery of gastrointestinal motility than without Dex (Chen et al., 2016). Although Dex is usually administered with a loading dose followed by a maintenance dose, this regimen can lower heart rate. Therefore, anesthesiologists prefer administering Dex intravenously with a loading dose followed by a maintenance dose or only as a maintenance dose during anesthesia and surgery. Based on these findings, we speculated that a relatively high dose of Dex with loading and maintenance doses may negate the pathogenesis of POGD than a maintenance dose only and, therefore, can alleviate intestinal injury and reduce POGD severity. Hence, we conducted this retrospective study, which was in accordance with the STrengthening the Reporting of OBservational studies in Epidemiology (STROBE), to assess if the administration of a loading plus maintenance dose of Dex during surgery causes better postoperative gastrointestinal recovery than the use of its maintenance dose only.

## Patients and Methods

### Design and Patients

This retrospective, single-center, cohort study was the subsequent analysis of a previous clinical study (NCT03086304). Patients who underwent elective colorectal surgery at the First Affiliated Hospital of Xi’an Jiaotong University from October 2017 to December 2019 were included in the present study. The study received ethical approval from the Ethics Committee of the First Affiliated Hospital of Xi’an Jiaotong University (Approval No. XJTU1AF2016LSL-035). Data were obtained from electronic and paper records and by telephone follow-up. Patients were divided into the loading plus maintenance dose (LMD) group and maintenance dose only (MD) group according to whether they received a loading dose of Dex or not, respectively. The LMD group was infused with a Dex loading dose of 1 μg/kg for 10 min followed by a maintenance dose of 0.4 μg/kg/h for continuous infusion. The MD group was only given a maintenance dose of 0.4 μg/kg/h. The inclusion criteria were as follows: 1) age ≥18 years with American Society of Anesthesiologists (ASA) grade I–III; 2) underwent selective colorectal surgery under general anesthesia; and 3) used Dex in general anesthesia. The exclusion criteria were as follows: 1) use of intraoperative enterostomy; 2) unclassified Dex administration dose or mode; or 3) history of intestinal surgery.

### Exposure and Outcomes

All patients received pre-medications of 0.075 mg palonosetron, 5 mg dexamethasone, and 0.5 mg penehyclidine hydrochloride. The LMD group was initially given 1 μg/kg Dex for 10 min, and then, the dose was maintained at 0.4 μg/kg/h until the end of the operation. In comparison, the MD group was only infused with the maintenance dose of Dex throughout the surgery. After transverse abdominis plane block (TAPB; 0.375% ropivacaine 20 ml) was carried out, all patients were given 0.5 μg/kg sufentanil, 0.2 mg/kg etomidate, and 1 mg/kg rocuronium for anesthesia induction and then administered with propofol, remifentanil, and rocuronium to maintain anesthesia. Their doses were adjusted based on the bispectral index of anesthesia depth, circulatory parameters, and muscular relaxation. The surgical options, such as colectomy or proctectomy, laparoscopy or laparotomy, postoperative diet, and treatment, were made by surgeons as routine clinical practice. Intraoperative fluid management was mainly adjusted according to the vital signs and arterial blood gas results of patients. All patients in the two groups received the identical postoperative intravenous analgesia pump formula, that is, sufentanil 100 μg and dexamethasone 10 mg diluted to 100 ml with normal saline. POGD diagnosis was defined as the absence of flatulence or defecation within 3 days after surgery accompanied by symptoms such as nausea, vomiting, and abdominal distension. The primary outcome was the first postoperative flatus time, and the secondary outcomes were the first postoperative defecation time and the incidence rates of abdominal distension, abdominal pain, nausea, and vomiting within 3 days postoperatively. In our previous clinical study (Gao et al., 2021), the patients and their caregiving family members were required to accurately record the first time of occurrence of the symptoms mentioned above by filling out the patient assessment forms, which would be transcribed on an evaluation sheet in the case report form (CRF) by researchers. If the patient did not have flatus within 3 days after the operation, the researchers would continue to follow-up by telephone until the first flatus time was obtained. Abdominal pain was defined as a Visual Analogue Scale (VAS) greater than 3 (0, no pain; 10, severe pain) within 3 days after the operation. Abdominal distension was divided into five grades from 1 (no at all) to 5 (very) according to subjective feeling. When the degree was greater than 3, the patient was considered having abdominal distension.

### Dex Plasma Concentration Simulation

The Dex pharmacokinetics of all patients from the beginning of Dex infusion to 10 h postoperatively was simulated by Tivatrainer software (version 9.1, Digital River GmbH Scheidtweilerstr, Cologne, Germany) according to the patients’ gender, age, height, weight, and ASA grade. The Dex plasma concentrations of all patients at the following time points were recorded: at 10 min, 30 min, 1 h, and 2 h post-administration; at the end of surgery; and at 2, 4, 6, 8, and 10 h postoperatively. The mean and standard deviation of plasma concentration in each group at each time point were calculated, and a pharmacokinetics chart was constructed using Origin software (Origin Lab Corporation, Northampton, America).

### Blood Sample Analysis

The blood samples obtained from a previous clinical trial (NCT03086304) were collected and centrifuged at 4°C, and the plasma was stored at −80°C. The blood samples were harvested at 24 h postoperatively. The samples from both groups with matching propensity scores were chosen for further analyses. The preoperative and interoperative variables that had the potential to influence POGD were selected based on univariate analysis and clinical impact (e.g., gender, age, presence of chronic obstructive pulmonary disease, operation method, tumor location, and anesthesia duration) (Bragg et al., 2015; Ceretti et al., 2018). Each patient’s propensity score in the selected variables was calculated by logistic regression. Thirty paired patients were randomly selected; one patient in the LMD group was matched with one patient in the MD group. Thirty paired blood samples were obtained at 24 h postoperatively from the paired patients. Plasma TNF-α, COX-2, DLA, Ach, and IL-10 levels were determined using enzyme-linked immunoassay (ELISA) kits from Shanghai Lianmai Bioengineering Co., Ltd. (Shanghai, China), and plasma LPS, IL-6 and iNOS levels were determined using ELISA kits from Cusabio Biotech Co., Ltd. (Wuhan, China).

### Statistical Analysis

Continuous variables with normal distribution were described as mean (standard deviation, SD), whereas those with non-normal distribution were described as median (range interquartile, IQR). Categorical variables were presented as numbers (percentages, %). Continuous variables were analyzed by Student’s t-test, and categorical variables were analyzed by the chi-square test and Fisher’s exact test as appropriate. The non-parametric Mann–Whitney *U* test was used for the comparison of ranked data. The role of Dex was further verified by calculating the risk factors of POGD through logical regression and conducting stratification and interaction tests on the remarkably different factors between the LMD and MD groups. All statistical analyses were performed in R version 3.1.2 (R Foundation for Statistical Computing, Vienna, Austria) and Empower Stats software (X&Y Solutions, Inc., Boston, Massachusetts). *p* < 0.05 was considered statistically significant.

## Results

### Perioperative Characteristics

**FIGURE 1:**
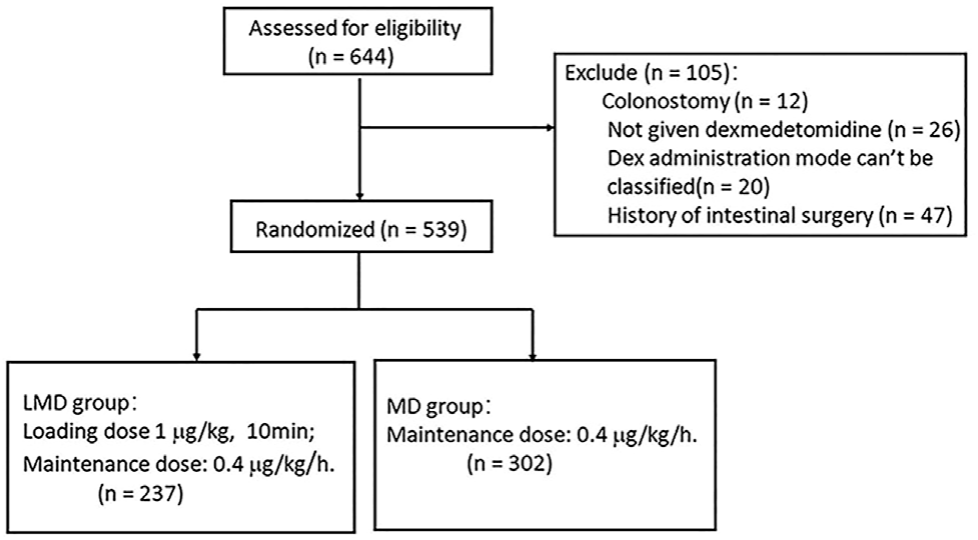
The research flow chart of 644 colorectal cancer patients recruited from October 2017 to December 2019.

**FIGURE 2:**
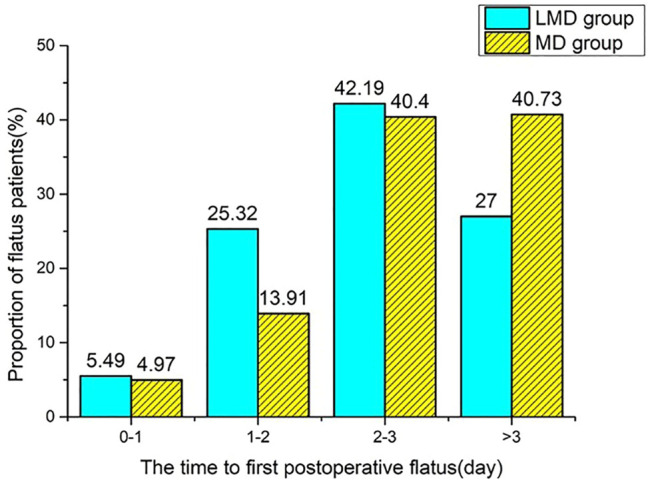
The proportion of flatus patients at each time point (less than or equal to 1 day, denoted as 0–1; greater than 1, less than or equal to 2 denoted as 1–2; greater than 2, less than or equal to 3 denoted as 2–3). Compared with the MD group, the LMD group had more patients with the first flatus time less than 3 days (73.00% vs. 59.28%), *p* < 0.05.

Among the 644 patients who met the inclusion criteria, only 618 patients (95.95%) were administrated with Dex. We excluded 12 who had a colostomy, 26 without Dex infusion, 20 whose Dex administration mode cannot be classified, and 47 with a history of intestinal surgery. A total of 539 patients were included in this study. Among them, 237 patients (38.35%) were given loading plus maintenance dose (LMD group) and 302 patients (48.87%) had an infusion of maintenance dose only from anesthesia induction (MD group, Figure 1). The two groups had no statistical difference in general demographic characteristics, comorbidity, and tumor characteristics (Table 1). The intraoperative anesthesia, postoperative analgesia, surgery, and vital sign variables are presented in Table 2 and Supplementary Table S1. There was no significant difference in the dosage of opioid drugs for postoperative analgesia and the proportion of patients receiving TAPB between the two groups (95.83 ± 13.54 vs. 94.27 ± 15.95, *p* = 0.231; 32.07% vs. 35.43%, *p* = 0.413). The LMD group had more patients who underwent laparoscopic surgery and proctectomy than those in the MD group and had lower heart rates at the end of loading dose infusion (T1). As shown in Table 3, the LMD group also had remarkably shorter flatus time and lower incidence rates of nausea, vomiting, abdominal distension, and abdominal pain compared to the MD group. The incidence of POGD was lower in the LMD group than in the MD group (27.00% vs. 40.40%, *p* = 0.002). The proportion of patients who had flatulence within 3 days postoperatively was higher in the LMD group than in the MD group (73.00% vs. 59.28%, *p* = 0.037, Figure 2).

**TABLE 1:** General demographic characteristics.

Variable	LMD group [*n* = 237 (43.97%)]	MD group [*n* = 302 (56.03%)]	P
Gender (F/M)	99/138	139/163	0.324
Age (years)	61.22 ± 11.60	59.90 ± 12.15	0.205
Weight (kg)	62.64 ± 10.49	62.45 ± 10.49	0.834
Height (cm)	165.97 ± 7.18	165.44 ± 7.34	0.402
BMI	22.70 ± 2.94	22.73 ± 3.04	0.906
ASA grades, n (%)			0.621^&^
Ⅰ	14 (5.9)	18 (6.0)	
Ⅱ	189 (79.7)	246 (81.5)	
Ⅲ	34 (14.3)	38 (12.5)	
COPD, n (%)	8 (3.38)	12 (3.97)	0.715
Coronary heart disease, n (%)	9 (3.8)	16 (5.3)	0.411
Diabetes, n (%)	26 (11.0)	27 (8.9)	0.432
Hypertension, n (%)	71 (30.0)	71 (23.5)	0.092
Tumor size			0.094^#^
>5 cm, n (%)	51 (21.5)	84 (27.8)	
≤5 cm, n (%)	186 (78.5)	218 (72.2)	
Depth of infiltration, n (%)			0.164^*^
T0	14 (5.9)	12 (4.0)	
Tis	4 (1.7)	0 (0)	
T1	9 (3.8)	13 (4.3)	
T2	39 (16.5)	40 (13.2)	
T3	74 (31.2)	104 (34.4)	
T4	97 (40.9)	133 (44.0)	
Lymph node metastasis, n (%)			0.058^*^
N_0_	157 (66.2)	194 (64.2)	
N_1_ (1–3)	46 (19.4)	68 (22.5)	
N_2_ (≥4)	34 (14.3)	34 (11.3)	
N_3_	0	5 (1.7)	
Distant metastasis, n (%)			0.676^#^
Yes	10 (4.2)	15 (5.0)	
No	227 (95.8)	286 (94.7)	

Values are presented as N (column %) or Mean ± SD. ^&^Mann–Whitney *U* test; ^#^chi-square test; *Fisher’s exact test.

ASA, American Association of Anesthesiologists; BMI, body mass index; COPD, chronic obstructive pulmonary disease.

**TABLE 2:** Indicators related to surgical and anesthesia.

Variable	LMD group (*n* = 237)	MD group (*n* = 302)	P
Operative method, n (%)			<0.01^a^
Laparoscopic	189 (79.7)	184 (60.9)	
Open surgery	48 (20.3)	118 (39.1)	
Operation time (min)	208.55 ± 66.45	201.29 ± 66.50	0.211
Anesthesia time (min)	239.85 ± 69.97	228.90 ± 70.22	0.073
Extubate time (min)	47.93 ± 24.36	46.79 ± 26.52	0.610
Loading dose of Dex (μg)	62.64 ± 10.49	0	<0.01^*^
Maintenance dose of Dex (μg)	100.64 ± 37.83	96.05 ± 36.63	0.155
General anesthetic			
Propofol (mg)	985.35 ± 403.83	958.43 ± 367.21	0.420
Sufentanil (μg)	30.68 ± 6.37	30.31 ± 6.74	0.522
Remifentanil (mg)	1.53 ± 0.55	1.58 ± 0.52	0.307
Rocuronium (mg)	86.44 ± 16.78	84.70 ± 16.24	0.225
Total infusion	2,646.29 ± 646.96	2,679.64 ± 683.33	0.565
Colloidal (ml)	872.29 ± 287.29	847.49 ± 317.63	0.347
Crystal (ml)	1725.74 ± 571.76	1803.54 ± 549.70	0.110
Blood products, n (%)	27 (11.39)	35 (11.59)	0.943
Blooding loses (ml)	138.43 ± 113.19	142.30 ± 113.53	0.694
Urine volume (ml)	784.68 ± 500.55	726.19 ± 487.08	0.173
Postoperative analgesia			
Sufentanil (μg)	95.83 ± 13.54	94.27 ± 15.95	0.231
TAPB, n (%)	76 (32.07)	107 (35.43)	0.413
Colorectal resection, n (%)			0.024^a^
Colectomy	136 (57.4)	202 (66.9)	
Proctectomy	101 (42.6)	100 (33.1)	

a Chi-square test, *independent-sample *t*-test.

Values are presented as N (%) or Mean ± SD.

TAPB, transversus abdominis plane block.

**TABLE 3:** Patient recovery index.

Variable	LMD group [N = 237 (43.97%)]	MD group [N = 302 (56.03%)]	Mean difference (95%CI)	*P*
Flatus time (day)	3.08 ± 1.21	3.56 ± 1.53	−0.487 (−0.719, −0.255)	<0.01^a^
Defecation time (day)	4.85 ± 2.68	4.89 ± 2.38	−0.042 (−0.486, −0.402)	0.852
Incidence of POGD, n (%)	64 (27.00)	122 (40.40)		0.002
Nausea, n (%)	60 (25.3)	102 (33.8)	—	0.034
Vomiting, n (%)	33 (13.9)	70 (23.18)	—	0.007
Abdominal distension, n (%)	175 (73.8)	296 (98.0)	—	<0.01
Abdomen pain, n (%)	86 (32.3)	141 (46.7)	—	0.002
Incidence of POGD, n (%)				
LOS (day)	17.45 ± 4.81	17.61 ± 5.83	-0.162 (-1.083, -0.759)	0.727

a Independent-sample *t*-test.

Values are presented as Mean ± SD or N (%).

LOS, length of stay.

### Logistic Regression Analysis of Dex and POGD

Compared with the MD group, the LMD group had an extra Dex loading dosage of 62.63 ± 10.51 μg, more laparoscopic surgery, and more proctectomy (Table 2). The LMD group was an independent protective factor of POGD (OR = 0.59, 95% CI = 0.41–0.87, *p* = 0.007), but the operation method and colorectal resection were not independent risk factors (Table 4). After stratified analysis, the protective performance of the LMD group had no statistical difference in each layer (*P* for interaction: operation method, 0.968; colorectal resection, 0.605). The LMD group showed the significant protective effect of laparoscopy (OR = 0.60; 95% CI = 0.384–0.929, *p* = 0.022) and colectomy (OR = 0.52, 95% CI = 0.322–0.828, *p* = 0.006).

**TABLE 4:** Logistic regression and subgroup analysis of LMD and POGD.

	N	Or (95%CI)	*P*	*P* for interaction
Operation method	539	0.69 (0.47–1.02)	0.066	—
Colorectal resection	539	0.97 (0.67–1.41)	0.866	—
LMD	539	0.59 (0.41–0.87)	0.007	—
LMD subgroup analysis				
Laparoscopic				0.986
Yes	373	0.60 (0.38–0.93)	0.022	
No	166	0.59 (0.29–1.19)	0.143	
Colectomy				0.605
Yes	338	0.52 (0.32–0.83)	0.006	
No	201	0.63 (0.35–1.14)	0.124	

### DEX Plasma Concentration–Time Course Curve and Serological Indexes of POGD

**FIGURE 3:**
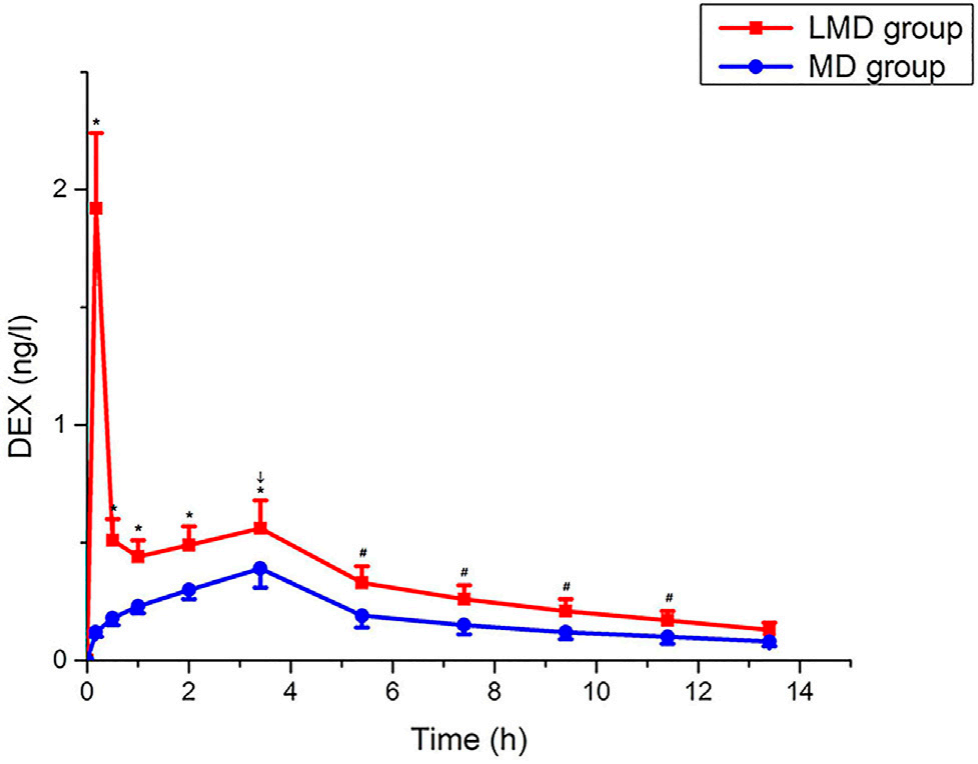
Change trend of Dex plasma concentration. The plasma concentration of the LMD group was significantly higher than that of the MD group, which lasted until 8 h after the operation*, p* < 0.05. “↓”: end of the operation. “*”: blood drug concentration at each time point during operation. “#”: the plasma concentration at 2, 4, 6, and 8 h after the operation.

The plasma Dex concentration in the LMD group was remarkably higher than that in the MD group during operation (Figure 3) until 8 h postoperatively. The LMD group had two peaks of plasma Dex concentration. The first peak appeared at the end of load infusion, and the peak concentration (1.922 ± 0.323 ng/L) was much higher than that (0.124 ± 0.026 ng/L, *p* < 0.01) in the MD group at the corresponding time point. The second peak appeared at the end of the operation (0.564 ± 0.122 ng/L vs. 0.391 ± 0.083 ng/L for the LMD and MD groups, respectively; *p* < 0.01).

**FIGURE 4:**
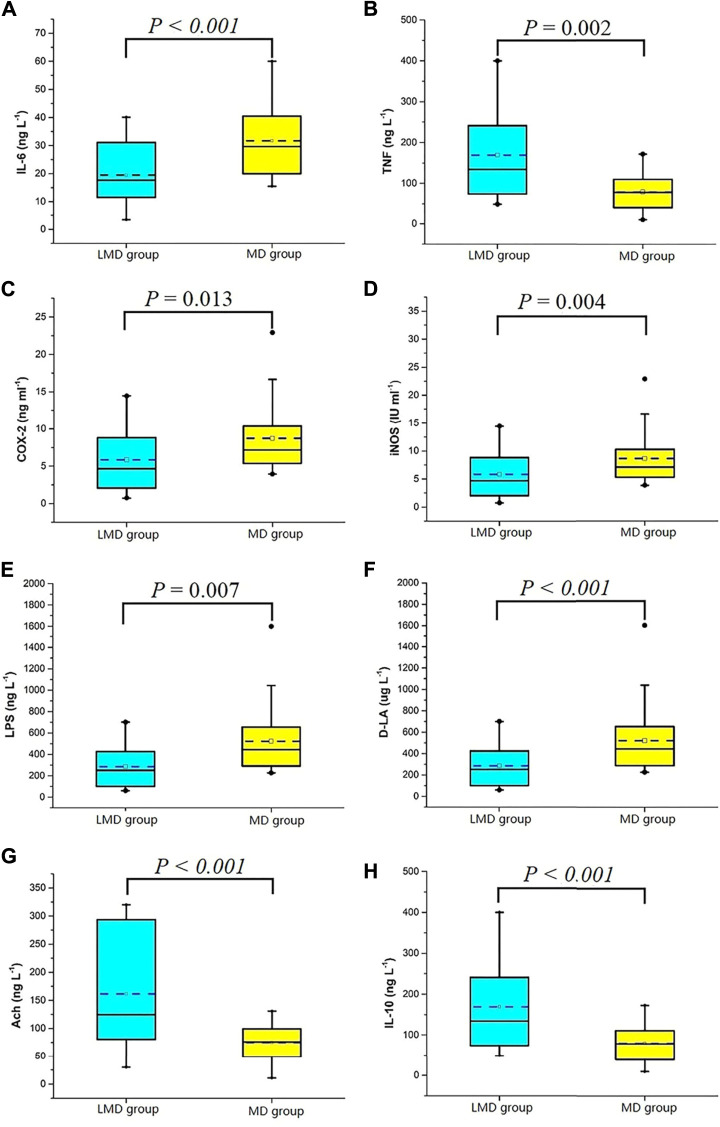
Serological indexes 24 h after the operation. **(A)** IL-6, interleukin-6; **(B)** TNF, tumor necrosis factor; **(C)** COX-2, cyclooxygenase-2; **(D)** iNOS, inducible nitric oxide synthase; **(E)** LPS, lipopolysaccharide; **(F)** D-LA, D-lactate; **(G)** Ach, acetylcholine; **(H)** IL-10, interleukin-10.

The biomarkers of the two groups were matched by propensity score, and there was no significant difference in confounding factors such as operation mode and resection site between the groups. Yet, the first flatus time of the LMD group was shorter than that of the MD group (Table 5). The two groups had significant differences in biomarkers at 24 h postoperatively (Figure 4). The LMD group had higher Ach (median [IQR]:124.5 [79.6, 293.6] ng/L vs. 75.7 [48.7, 100.9] ng/L, *p* = 0.000) and IL-10 concentrations (134.2 [73.8 242.4] ng/L vs. 76.7 [37.5, 111.5] ng/L, *p* < 0.001) and iNOS (7.4 [4.2, 12.0] IU/ml vs. 12.1 [7.3, 19.6] IU/ml, *p* = 0.004), COX-2 (4.7 [2.0, 9.0] ng/ml vs. 7.2 [5.3, 10.5] ng/ml, *p* = 0.013), IL-6 (17.7 [11.5, 31.8] ng/L vs. 29.7 [19.9, 40.7] ng/L, *p* = 0.000), TNF-α (301.7 [108.1, 400.0] ng/L vs. 400.0 [332.5, 400.0] ng/L, *p* = 0.002), LPS (37.0 [16.5, 59.5] ng/L vs. 59.7 [31.4, 89.2] ng/L, *p* = 0.007), and DLA concentrations (250.3 [98.6, 427.9] μg/L vs. 443.7 [289.7, 657.2] μg/L, *p* < 0.001) than the MD group.

**TABLE 5:** Characteristics of paired samples.

Variable	LMD group (*n* = 30)	MD group (*n* = 30)	*P*
Gender (F/M)	16/14	15/15	0.796
Age (years)	59.67 ± 11.17	58.90 ± 8.30	0.764
COPD, n (%)	4 (13.3)	3 (10.0)	0.688
Anesthesia time (min)	250.17 ± 82.50	235.33 ± 61.31	0.432
Operation time (min)	219.83 ± 77.19	209.50 ± 59.95	0.565
Laparoscopic			0.176^a^
Yes, n (%)	22 (73.3)	17 (56.67)	
No, n (%)	8 (26.67)	13 (43.3)	
Colectomy			0.284^a^
Yes, n (%)	17 (56.67)	21 (70.0)	
No, n (%)	13 (43.3)	9 (30.0)	
Flatus time (day)	3.13 ± 1.33	3.87 ± 1.38	0.041
Defecation time (day)	4.70 ± 2.23	5.25 ± 2.44	0.374

a Chi-square test.

Values are presented as Mean ± SD or N (%).

COPD, chronic obstructive pulmonary disease.

## Discussion

The current retrospective study found that Dex use with an extra loading dose enhanced the recovery of gastrointestinal function and decreased the biomarkers of intestinal barrier injury. Indeed, the extra loading dose of Dex remarkably reduced the postoperative flatus time by 0.5 days, as well as the incidences of postoperative nausea, vomiting, abdomen pain, and abdominal distension. The favorable effects of the extra Dex dose may be due to the higher Dex concentration in the blood from skin incision until 8 h postoperative, which likely lowered sympathetic tone, increased parasympathetic tone, and inhibited inflammation. The side effect of the extra loading dose of Dex was a lower heart rate, which was within the physiological range and likely had no clinical significance.

POGD is a transient gastrointestinal impairment after an abdominal surgery that compromises postoperative recovery. Current treatment strategies only limit the symptoms of POGD. Dex is widely used clinically during surgery, relieves intestinal inflammation, and protects the intestinal barrier in the intestinal ischemia–reperfusion rat model (Zhang et al., 2012). Our two groups of patients received Dex during surgery but with different administration modes and Dex dosages. The relatively higher dose of Dex was likely responsible for the favorable effects found in this study. Indeed, the patients in the LMD group had a larger dose than those in the MD group. Hence, the higher pharmacodynamics and longer pharmacokinetics may induce more drug effects against the occurrence of POGD, although more laparoscopic surgery and rectal tumor in the LMD group may also contribute to the less occurrence of POGD (Table 2). However, the logistic regression analyses showed that the extra dose of Dex but not laparoscopic surgery and colectomy was an independent protective factor (OR = 0.59; 95% CI = 0.41–0.87, *p* = 0.007). Laparoscopic surgery promotes the recovery of gastrointestinal function because of its minimal invasion (Wang et al., 2014). The proportional use of laparoscopic surgery in both groups was also comparably high; therefore, laparoscopy was not an independent protective factor (OR 0.69; 95% CI 0.47–1.02; *p* = 0.066). Our study was in line with a previous study, which reported that colectomy and proctectomy are not independent risk factors (Millan et al., 2012). Our stratified analysis further indicated that the extra loading dose of Dex was an independent protective factor.

Surgical operation activated the neurogenic stage of POGD from skin incision to 3–6 h postoperatively, in which the inhibition of vagus nerve tone widely inhibited gastrointestinal motility (Bauer and Boeckxstaens, 2004; Bragg et al., 2015; Stakenborg et al., 2017; Wattchow et al., 2021). The extra loading dose of Dex had a 4.9-fold higher peak concentration than without this dose at the end of the surgery, and its effects lasted longer up to 8 h postoperatively, which covered the neurogenic stage of POGD. This extra dose also increased plasma Ach level up to 24 h operatively and, therefore, can enhance vagus nerve tone.

Continuous intestinal inflammation prolongs the excessive excitement of the intestinal sympathetic nerve and inhibits gastrointestinal peristalsis (Bauer and Boeckxstaens, 2004; Wattchow et al., 2021). The inflammatory stage of POGD covers from 3 to 4 h intraoperatively to 72 h postoperatively (Stakenborg et al., 2017). Surgical manipulation activates macrophages in the intestinal wall; activates transcription factors, such as nuclear factor κB; causes the release of pro-inflammatory mediators (IL-6, TNF-α, COX-2, and iNOS); causes neutrophil and monocyte infiltration into the intestinal muscle layer; and releases nitric oxide and prostaglandin (Wang et al., 2018). It also destroys the pacing activity of interstitial cells of Cajal and damages the intestinal barrier (LPS and DLA) (Funder et al., 2017; Kaji et al., 2018). Previous reports suggested that Dex decreases inflammatory responses in different animal models and different types of surgeries (Li et al., 2015; Yeh et al., 2016; Wang and Li, 2018). Our study showed that the extra dose of Dex caused a higher Dex plasma concentration from skin incision up to 8 h postoperative, which is also the time for POGD development. Therefore, the higher Dex dose inhibited the pathogeneses of POGD. The extra Dex dose also induced an increase in plasma IL-10 levels but decreased IL-6, TNF-α, COX-2, iNOS, LPS, and DLA levels. The anti-inflammatory effect and the enhancement of vagus nerve tone caused by Dex ultimately promoted intestinal function recovery after surgery.

The strength of our study is the relatively large sample size and the modulation of the pathogeneses of POGD using a higher dose of Dex with favorable changes in biomarker levels, which is the first report in this setting. However, this study is a retrospective one, and as such, many factors cannot be controlled; hence, the findings of the current study require further studies, including clinical trials.

**FIGURE 5:**
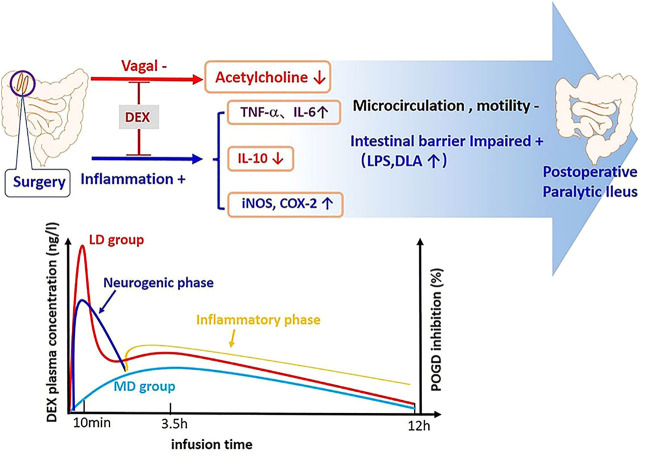
Pathogenesis of POGD and mechanism of intestinal protection by Dex. There are two phases of POGD: the neurogenic phase and inflammatory phase. The extra dose of Dex caused a higher level of Dex plasma concentration from the skin incision to postoperative 8 h, and this period of time is also the time for the POGD development. In the meantime, Dex also induced significantly higher plasma IL-10, but lower IL-6, TNF-α, COX-2, iNOS, LPS, and DLA.

In conclusion, the extra loading dose of Dex superposed with its maintaining dose can increase parasympathetic tone, decrease inflammation, and hence, promote better postoperative gut function recovery. However, the extra loading dose caused a lower heart rate, which is likely is not clinically significant (Figure 5). Therefore, the loading and maintaining administration modes of Dex should be considered for clinical use, but further studies are needed.

## Data Availability

The original contributions presented in the study are included in the article/Supplementary Material; further inquiries can be directed to the corresponding author.
