# Supplementary material for: Extra Loading Dose of Dexmedetomidine Enhances Intestinal Function Recovery After Colorectal Resection: A Retrospective Cohort Study
Source: Front Pharmacol. 2022 Apr 25;13:806950. doi: 10.3389/fphar.2022.806950 (PMC9081762; doi:10.3389/fphar.2022.806950)
Supplement: Supplementary file 1 [file Table1.docx]

| Table 1. Description of the methods to assess adherence used on the included studies | | |
| --- | --- | --- |
| Author, Year | **Adherence methods** | **Adherence methods description** |
| Na-Bangchang et al, 1997 | Biological Assay | Blood Mefloquine concentrations on day 2 compared with reference profiles from hospitalized patients under supervisioned treament |
| Fungladda et al, 1998 | 1.Self-reported Measure 2. Pill count | 1. Interview on day 5 for AS and day 7 for QN+TET* 2. The blister pack was examined on day 5 for AS and day 7 for QN+TET for remaining tablets |
| Qingjun et al, 1998 | Self-reported Measure | Questionnaire applied on day 8* |
| Fogg et al, 2004 | 1.Self-reported Measure 2.Pill count 3.Biological assay | 1. Open questionnarie - a structured interview concerning the time and method of taking each dose, applied on day 3 2. The blister pack was examined on day 3 for remaining tablets  3. Blood Lumefantrine concentrations correlated with the results of indirect methods assessed* |
| Rocha, 2008 | 1.Self -reported Measure 2.Clinical cure | 1. Interview asking if the participant took the medication as prescribed by the healthcare professional and describing how it was taken applied on day 7 2. Absence of symptoms assessed on day 7 |
| Asante et al, 2009 | Pill count | The blister pack was examined on day 2 for remaining tablets |
| Lemma et al, 2011 | 1.Self-reported Measure 2.Pill count | 1. Questionnaire applied on day 3* 2. The blister pack was examined on day 3 for remaining tablets |
| Tun et al, 2012 | 1.Self-reported Measure 2.Pill count | 1. Questionnaire applied on day 3* 2. The blister pack was examined on day 3 for remaining tablets |
| Almeida et al, 2014 | 1.Self-reported Measure 2.Pill count | 1. A 5 item self-reported questionnarie adding one question to Morisky’s 4-item questionnaire (Dichotomous and Likert scale) applied on day 7 2. The blister pack was examined on day 7 for remaining tablets |
| Ferreira et al, 2014 | 1.Self-reported Measure 2.Pill count | 1. Interview with one question - "Could you take the prescribed medications?” applied on day 3 for *Pf* and day 6 for *Pv* 2. The blister pack was examined on day 3 for *Pf* and day 6 for *Pv* for remaining tablets |
| Minzi et al, 2014 | 1.Self-reported Measure 2.Pill count 3.Biological Assay | 1. Interview - A structured interview to determine how the regimen was taken, the time and method of taking each dose was then conducted, applied on day 3 2. The blister pack was examined on day 3 for remaining tablets  3. Blood Lumefantrine concentrations on day 7, that corresponds to 24 hours after 7 days of AL intake |
| Amponsah, 2015 | Pill count | The blister pack was examined on day 3 for remaining tablets |
| Cheoymang et al, 2015 | 1.Self-reported Measure 2.Pill count 3.Biological assay | 1. Interview without questionnaire applied on days 3, 7, and 14* 2. The blister pack was examined on days 3, 7, and 14 for remaining tablets 3. Blood Primquine concentrations collected about 2–4 h after dosing on days 3, 7, and 14 of the initial treatment for the determination of primaquine concentrations, describing the minimum, maximum and outliers of plasma concentrations |
| Osorio-de-Castro et al, 2015 | 1.Self-reported Measure 2.Pill count | 1. Interview applied on day 2 for *Pf* and day 5 for *Pv** 2. The blister pack was examined on day 2 for *Pf* and day 5 for *Pv* for remaining tablets |
| Souza et al, 2016 | 1.Self-reported Measure 2.Pill count | 1. Interview with one question: “Could you take the prescribed medications?” 2. The blister pack was examined for remaining tablets |
| Steury, 2016 | 1.MEMS 2.Pill count | 1. The MEMS cap on the pillbox containing the ACT electronically recorded the time of each opening of the medication bottle beginning with the first dose on day 3 to 1 week 2. The blister pack was examined on day 3 to 1 week for remaining tablets |
| Saravu et al, 2018 | 1.Self-reported Measure 2.Pill count | 1. Interview applied on day 6* 2. The blister pack was examined on day 6 for remaining tablets |
| Takahashi et al, 2018 | 1.Self-reported Measure 2.Pill count | 1. Interview (home visit or telephone) applied on day 3 or 4* 2. The blister pack was examined on day 3 or 4 for remaining tablets |
| Oduro et al, 2019 | 1.Self-reported Measure  2.Pill count | 1. Interview - The in-depth interview included a day-by-day account of the number of doses taken, number of tablets taken during each dose, time of each dose, reasons for any leftover or missed dose, and whether or not there was vomiting, applied on day 3 2. The blister pack was examined on day 3 for remaining tablets |
| Bagchi et al, 2020 | 1.Self-reported Measure 2.Self-reported Measure 3.Pill count | 1. Interview applied on day 3* 2. Subject’s self-reported compliance asked on day 3* 3. The blister pack was examined on day 3 for remaining tablets |
| Rosa et al, 2020 | 1.Self-reported Measure | 1. Morisky Medication Adherence Scale (MMAS-8) questionnaire |
| *The authors did not provide brief a description of the method. ACT - Artemisinin-based Combination Therapy; AL - Artemether + Lumefantrine; AS - Artesunate; MEMS - Medication Event Monitoring System; *Pf* – Plasmodium falciparum; *Pv* – Plasmodium vivax; QN - Quinine; TET – Tetracycline. | | |
